# Remarks on Leeches

**Published:** 1858-12

**Authors:** Frederick Stearns


### REMARKS ON LEECHES.

BY FREDETICK STEARNS.

A CONSTANT supply of healthy and vigorous foreign varieties of these animals is considered, by practitioners in those districts where they can be afforded cheaply, to be an invaluable aid in practice. The cheapening of them in this country, where they have hitherto borne so high a price, we must consider no small benefit to the community. To the enterprise and experience of Mr. Witte, of New York, (prominent in that market for the past twenty-five years as an importer of leeches,) is due the credit for first accomplishing the above benefits, as he now offers, through his own house and those of his agents, fresh and healthy Swedish and Hungarian leeches, at prices which will bring them within the reach of all requiring their aid.

In view of an increased use of leeches in the interior, where they are now employed to a limited extent only, it has been suggested to us to state, for the benefit of our readers who may not be familiar with their use, the most practicable methods of preserving and applying them.

Leeches are sent out by dealers usually in boxes filled with marsh-sod and clay. It is best that they should be kept in this; all the care they then require is to occasionally moisten the earth, and remove the dead or sickly leeches, if there be any. The box should be kept in a moderately cool cellar. If more convenient to keep them in water, a suitable jar should be provided, in the cover of which are fine perforations, to allow of a circulation of air. Rain-water should be employed, and changed daily in summer, though less often will answer in winter, always observing to remove dead and sickly ones, in order to preserve the rest, as the diseases affecting leeches are mostly epidemic in their character.

It is necessary, in applying leeches, that the part to be leeched should be perfectly clean and free from smell of medicine or perspiration. Handle the leeches always carefully, and with clean hands. Having determined upon the number to be used, place them in a cupping-glass, wine-glass, or even in the bottom part of a chip pill-box, and invert it over the part affected. It is often desirable to partly fill the cupping-glass with water, as they will bite more readily when covered by it. When they have attached themselves, the cup can be gently removed, and the part surrounded by a soft cloth, which will absorb the moisture and blood, and catch the leeches when they drop off.

It often occurs that leeches, from some unknown cause, can not be made to bite by ordinary means. We have found that with such, the best success attends a slight scarification of the affected part, and the subsequent application of the leech, by holding it in a soft dry towel, and directing its head to the scarified part, withdrawing it a little as it reaches it, thus compelling it to fix thereat. Vinegar, milk, molasses, etc., are useless and unnecessary to incite leeches to bite.

For the application of leeches to the gums, and to the neck of the uterus, tubes are made, by means of which the leech is compelled to attach itself to any spot desired.

Pereira states that "several circumstances affect the fixing of leeches; as the condition of the animal, whether healthy or otherwise; the nature or condition of the part to which it is applied: thus, leeches will not readily attach themselves to the soles of the feet or the palm of the hands, or to the hairy parts—the presence of grease, vinegar, salt, and some other substances will prevent them from biting. Durheims says, leeches will not bite those under the influence of sulphur, on account of the evolution of sulphureted hydrogen by the skin. The effluvia or vapors of the room, as the fumes of tobacco, sulphur, vinegar, will prevent their biting, or even cause them suddenly to fall off."

If a flow of blood greater than that swallowed by the leech is desired, it must be excited, after the leech drops off, by warm fomentations or poultices.

The flow of blood from leech-bites is best checked by compression with lint, or the introduction of cone-shaped plugs of it into the wound, by means of a probe; the usual hæmostatics may also be used.

It is not recommended that leeches once used be preserved; but if they are kept for future use, they should not be stripped between the fingers nor placed upon salt, (the first kills them outright, and the second blisters them,) but they should be placed in a jar, apart from those not used, changing the water occasionally, and when they have digested the blood they are gorged with, they will generally bite readily again.

It is thought by many that the bite of a leech once used for bleeding a person suffering of an infectious disease may be the means of communicating it to others.—*Peninsular Medical Journal.*

